# Revision of *Coprosma* (Rubiaceae, tribe Anthospermeae) in the Marquesas Islands

**DOI:** 10.3897/phytokeys.4.1600

**Published:** 2011-07-12

**Authors:** Warren L. Wagner, David H. Lorence

**Affiliations:** 1 Department of Botany, MRC-166, National Museum of Natural History, Smithsonian Institution, P.O. Box 37012, Washington, DC 20013-7012; 2 National Tropical Botanical Garden, 3530 Papalina Road, Kalaheo, HI 96741 USA

**Keywords:** Conservation, *Coprosma*, French Polynesia, Marquesas Islands, Rubiaceae

## Abstract

During the preparation of the Vascular Flora of the Marquesas Islands three new species of *Coprosma* (Rubiaceae, tribe Anthospermeae) have come to light and are described herein: *Coprosma fatuhivaensis* W. L. Wagner & Lorence, *Coprosma meyeri* W. L. Wagner & Lorence, and *Coprosma temetiuensis* W. L. Wagner & Lorence. Descriptions, illustrations, conservation status, and specimen citations are provided. Amended descriptions of three previously described Marquesan *Coprosma* species are also provided as well as a key to the species, four of which fall into the Critically Endangered (CR) and two into the Endangered (EN) category. With the description of these the new species, *Coprosma* becomes the sixth largest lineage in the Marquesas Islands with six species after *Psychotria* (one lineage which has 9 spp.), *Cyrtandra* (8 spp.), *Bidens* (8 spp.), *Melicope* (7 spp.), and *Ixora* (7 spp.).

## Introduction

*Coprosma* J.R. Forst. & G. Forst.is a genus of about 110 species, unusual in most species being dioecious and wind-pollinated. The genus is widely distributed on Pacific islands with a primary center of diversity in New Zealand (ca. 50 spp.), but with secondary centers of diversity the Hawaiian Islands (13 spp.), New Guinea (11 spp.), and Australia (8 spp.). The remainder are scattered over a wide area of the Pacific Basin, extending to southeastern Polynesia and the Juan Fernández Islands. There are 16 species of *Coprosma* in Polynesia, with six species endemic to the Marquesas Islands, four in the Society Islands, three in the Australs, two in Samoa, and one each in the Tuamotu Islands, Pitcairn Island, and Cook Islands.

Oliver (1935) divided the genus into seven groups, most of which were subdivided into smaller groups of presumably closely related species. He placed all southeastern Polynesia species then known into his *Coprosma pyrifolia* group characterized by relatively large leaves that are usually obovate to ovate and finely reticulate, triangular stipules, which are entire to denticulate, male flowers in small clusters with a calyx present, and female flowers 3 per cluster, the calyx lobes as long as or shorter than the tube. He hypothesized that this group was related to similar species in New Zealand. Florence (1986) in a paper describing two new Marquesan species (*Coprosma nephelephila* Florenceand *Coprosma reticulata* Florence) allied them and the one other Marquesan species then known [*Coprosma esulcata* (F. Br.) Fosberg]with the orange-fruited Hawaiian species. Heads (1996) supported Florence's hypothesis by placing the Marquesan species in a group with the Hawaiian species, rather than the *Coprosma pyrifolia* group where all of the other southeastern Polynesian species were placed. No comprehensive study of relationships has been made, but a recent molecular study of Tribe Anthospermeae (Anderson et al. 2001), in which 6 of 16 of the taxonomic groups recognized by Heads (1996) were sampled, indicates an apparent Australian origin of *Coprosma* and possible independent colonization of Fiji and Hawaiian Islands from New Zealand. The two subgenera of *Coprosma* recognized by Heads (1996) were not supported. No Polynesian species were included in the sparse sampling of the genus leaving the relationships and biogeography of the genus in the Pacific completely open. The species in the following taxonomic section of this paper are arranged alphabetically.

Brown (1935) described the first species of *Coprosma* discovered in the Marquesas Islands in his Rubiaceae treatment for the *Flora of Southeastern Polynesia*, but mistakenly placed it in the genus *Psychotria* as *Psychotria esulcata* F. Br. Fosberg (1939) in his revision of Marquesan *Psychotria* maintained it as a species of *Psychotria*; however, he later (Fosberg 1956) realized it was actually a poorly understood species of *Coprosma* and transferred to its proper place as *Coprosma esulcata* (F. Br.) Fosberg. It remained the sole Marquesan member of the genus until the Flore de la Polynésie française project under the auspices of Jacques Florence at IRD (formerly ORSTOM) was initiated. Collecting in the Marquesas Islands intensified greatly with the initiation of this project and Florence (1986) discovered two additional species of *Coprosma* (*Coprosma nephelephila* and *Coprosma reticulata*). During the collecting expeditions for the current Vascular Flora of the Marquesas Islands project under the direction of David H. Lorence and Warren L. Wagner (Wagner & Lorence 1997) three additional species were discovered, bringing the total to six species. This paper, a generic revision for the Marquesas Islands with descriptions of new taxa, represents a precursor to the latter project.

## Conservation status

The Marquesan species of *Coprosma* are distributed with a typical higher diversity on the older and larger islands. Three species occur on Nuku Hiva (*Coprosma esulcata*, *Coprosma nephelephila* and *Coprosma reticulata*), one on Ua Pou (*Coprosma esulcata*), two on Hiva Oa (*Coprosma feaniana* and *Coprosma temetiuensis*), and one on Fatu Hiva (*Coprosma fatuhivaensis*). To date only 36 total collections of *Coprosma* have been made of the Marquesan species, which gives an indication of how uncommon these species are. Two of the species, *Coprosma esulcata* and *Coprosma reticulata*, constitute the bulk (26) of the collections. Both of these species occur as scattered individuals in appropriate habitats or are occasionally locally common. The other four species are known from only a few collections. When evaluated using the IUCN criteria for endangerment (IUCN 2001), four of the Marquesan *Coprosma* species of (*Coprosma fatuhivaensis*, *Coprosma meyeri*, *Coprosma nephelephila*, and *Coprosma temetiuensis* ) fall into the **Critically Endangered** (CR) category, which designates species facing the highest risk of extinction in the wild. Marquesan species of *Coprosma* meet the IUCN criteria by having known ranges less than 100 km2, an area of occupancy of less than 10 km2, continuing decline in the quality of habitat, and a populations size less than 50 mature individuals. *Coprosma esulcata* and *Coprosma reticulata* are considered **Endangered** (EN): B1, B2b (i–iii): B1 extent of occurrence <5,000 km²; B2: total area of occupancy less than 500 km² (c. 75 km²); B2b (i–iii), habitat continuing decline inferred in (i) extent of occurrence, (ii) areas of occupancy, and area, (iii) extent and/or quality of habitat. Further details are given under each species.

## Methodology

All measurements given herein are taken from dried herbarium specimens, although certain features such as shapes were supplemented with information from alcohol-preserved flowers and fruits, field notes, and color slides or digital photos. Measurements are presented in the descriptions as follows: length × width, followed by units of measurement (mm or cm). Specimens from the following herbaria were studied: BISH, K, MO, MPU, NY, P, PAP, PTBG, US, and WU). The area of occupancy (distribution) for each species was calculated using herbarium collection data and field observations, and the conservation status is proposed following the IUCN Red List Category criteria (IUCN 2001; www.iucnredlist.org/info/categories_criteria2001).

## Systematics

### Coprosma

J. R. Forster & G. Forster

http://species-id.net/wiki/Coprosma

#### Description.

*Shrubs*, multi-branched, erect, occasionally creeping and sometimes rooting at the nodes or *occasionally trees*, often foetid when bruised. *Leaves* simple, opposite or rarely ternate, margins entire, petiolate or sessile; stipules interpetiolar, distinct or partly connate, entire or dentate with toothlike marginal colleters. *Flowers* unisexual (and the plants dioecious or rarely monoecious), rarely polygamous or in one species perfect, axillary, solitary or in cymes; calyx 4–5(–10)-toothed, often reduced or absent in male flowers; corolla funnelform or campanulate, 4–5(–10)-lobed, lobes valvate in bud; stamens 4–5(–10), inserted at base of corolla tube; filaments long-exserted, erect or pendulous; ovary 2(–4)-celled, ovule 1 per cell, basal, anatropous; style 2(–4)-lobed, divided nearly to base; stigmas long-exserted, papillose-hirsute. *Fruits* drupaceous, juicy, ovoid to globose, with 2(–4), 1-seeded, plano-convex pyrenes.

#### Key to species of *Coprosma* in the Marquesas Islands.

**Table:** 

1a	Leaves ternate, chartaceous	4. *Coprosma nephelephila*
1b	Leaves opposite, thin- to thick-coriaceous	2
2a	Petioles 0–0.1 cm long; Leaf blades with secondary and higher venation obscure, only the midvein conspicuous	3. *Coprosma meyeri*
2b	Petioles 0.3–1.7 cm long; leaf blades with secondary and higher venation conspicuous, reticulate	3
3a	Leaves 0.9–1.8 cm wide, narrowly elliptic	2. *Coprosma fatuhivaensis*
3b	Leaves 2.0–6.0 (–13.0) cm wide, oblanceolate, oblong, or elliptic-oblanceolate	4
4a	Lower surface of leaves strigose along the veins; leaves thick-coriaceous	1. *Coprosma esulcata*
4b	Lower surface of leaves glabrous; leaves thin-coriaceous	5
5a	Leaves 5.1–9.1 × 2.1–3.6 cm; stipules 1.5–3 mm long	6. *Coprosma temetiuensis*
5b	Leaves 8.5–19.7 × 3–13 cm; stipules 3–5 mm long	5. *Coprosma reticulata*

### Coprosma esulcata

(F. Br.) Fosberg, Brittonia 8: 178. 1956.

http://species-id.net/wiki/Coprosma_esulcata

Psychotria esulcata F. Br. (Bernice P. Bishop Museum Bulletin 130: 315. 1935). [Basionym]

#### Type.

**Marquesas Islands**: Ua Pou: Without further locality, 1000 m, 1921, E. Qualye 1136(holotype: BISH-578803!).

#### Description.

*Shrub or small tree* 1.5–4 m tall; young stems glabrous. *Leaves* decussate, thick-coriaceous, blades 6.5–13 × 2.7–6 cm, ellipticoblanceolate, pinnately veined with 8–18 pairs of secondary veins, higher level venation conspicuously reticulate, upper surface glabrous, lower surface strigose along the veins, midrib broad, with a narrow wing, domatia small or sometimes apparently absent, located along midrib near juncture of secondary veins, apex acuminate, base cuneate; petioles 0.4–1.3 cm long, stout, narrowly winged; stipules ca. 3–8 mm long, connate 1/2–4/5 of length, both surfaces glabrous, margins ciliate with reddish brown hairs and dentate with conspicuous colleters, apex obtuse to a conspicuous appendage. *Inflorescences* axillary with 6(–15) flowers, trichotomously branched, with 1–3 nodes, the uppermost with a 3-flowered cymule, the others with usually only 1–2 flowers developing on each, these 5–6-merous, peduncles finely, sparsely strigulose. *Flowers:* male flowers with calyx campanulate, ca. 2 mm long, the tube 1 mm long, the lobes 1 mm long, corolla 6–7 mm long, the tube 5 mm long, the lobes ca. 2 mm long, staminal filaments 7 mm long; female flowers with peduncles 2–3 mm long, calyx tubular, 0.4–0.8 mm long, corolla narrowly funnelform, the tube 1.8–2 mm long, the lobes 1.4–1.8 mm long, the styles 9–11 mm long. *Fruits* ca. 6–7 mm long × 3 mm wide, obovoid-elliptic, ripening bright red or reddish orange, apex with persistent calyx teeth. *Pyrenes* obovoid-ellipsoid, ca 4 mm long × 2.5-3 mm wide hemispherical in cross-section, smooth, heavily slerified on the edges and on the flat inner face, thin on convex side.

#### Distribution.

Marquesas Islands, scattered to locally common on Ua Pou and a single collection known from Nuku Hiva.

#### Ecology.

This species is known from 770 to 920 m elevations on steep slopes or ridges in cloud-shrouded shrubland and wet forest dominated by *Freycinetia impavida* (Gaudich. ex Hombr.) B. C. Stone*, Pandanus tectorius* Parkinson, and *Metrosideros collina* (J. R. Forst. & G. Forst.) A. Gray.

#### Specimens Examined.

**Marquesas Islands:** **Nuku Hiva.** between Taiohae Bay and Hooumi Bay, 900 m, *Gagné 1159* (BISH, US). **Ua Pou:** Teavaituhai, 3000 ft, Mumford & Adamson 642 (BISH), Meyer 2835(PTBG, US); Teavahaakiti, steep slopes of main ridge to S of Oave, N & E facing cliffs between Teavahaakiti & Tekohepu, 2700 ft, Perlman & Wood 15905 (PTBG), 2550 ft, Perlman & Wood 15922 (PTBG, WU); Matahenua, between Oave and Poutetainui, high mountain peaks along main backbone ridge, 899 m, Perlman & Wood 19079(P, PAP, PTBG, US); forested ridge and slopes up to Teavahaakiti, northwest side, 914 m, Wood 10440 (PAP, PTBG, US), Wood 10446 (PTBG, US); central Ua Pou including the summit crest regions around Oave and the near-by peak of Matahenua., 2950-3030 ft, [09°23'454"S, 140°04'433"W], Wood & Perlman 10802 (PAP, PTBG, US); Tekohepo, summit, 2500-3000 ft, [09°24'31"S, 140°04'21"W], Wood & Perlman 6487 (PAP, PTBG), Wood & Perlman 6492 (PTBG).

#### Conservation status.

Following the criteria and categories of IUCN (2001) it is assigned a preliminary status of **Endangered** (EN): B1, B2b (i–iii): B1 extent of occurrence <5,000 km²; B2: total area of occupancy less than 500 km² (c. 50 km²); B2b (i–iii), habitat continuing decline inferred in (i) extent of occurrence, (ii) areas of occupancy, and area, (iii) extent and/or quality of habitat. The suitable habitat for *Coprosma esulcata* on Nuku Hiva (c. 340 km²) and Ua Pou (c. 105 km²) is indicated as an endangered environment, threatened by human activity (deforestation and fire), feral animals, and invasive plants, reducing the extent of the forest.

### Coprosma fatuhivaensis

W. L. Wagner & Lorence sp. nov.

urn:lsid:ipni.org:names:77112736-1

http://species-id.net/wiki/Coprosma_fatuhivaensis

Figs 1 4A, B 

#### Latin.

*Foliis tenuiter coriaceis, 5.3*–*6.7 cm longis* × *0.9*–*1.8 cm latis, anguste ellipticis, petiolis 0.7*–*1.1 cm longis, stipulis circa 2*–*2.5 mm longis.*

#### Type.

**Marquesas Islands**: Fatu Hiva:Tevaiua, southern summit region, 870 m, 15 February 2003, K. R. Wood 10137 (Holotype: PTBG!; Isotypes: AD!, BISH!, K!, MO!, MPU!, NY!, P!, PAP!, US!).

#### Description.

*Tree* ca. 7 m tall; young stems short-pilose. *Leaves* opposite, thinly coriaceous, blades 5.3–6.7 × 0.9–1.8 cm, narrowly elliptic, pinnately veined with 8–9 pairs of secondary veins, higher level venation conspicuously reticulate, both surfaces glabrous, domatia minute, usually subcircular, located along midrib near juncture of secondary veins, apex acute, base attenuate; petioles 0.7–1.1 cm long; stipules ca. 2–2.5 mm long, connate 4/5 of length, both surfaces glabrate, margins weakly ciliate and dentate with a larger and sometimes also a few small colleters, the colleters often with a small tufts of a few hairs. *Inflorescences* axillary, subsessile, with 3 flowers, these 5–6-merous. *Flowers:* male flowers unknown; female flowers with peduncles to 4.2 mm long, calyx short-tubular, ca. 0.5–0.6 mm long, the tube ca. 0.1–0.2 mm long, the lobes triangular, 0.3–0.4 mm long, corolla narrowly funnelform, the tube 2 mm long, the lobes 1.2–1.3 mm long. *Fruit and pyrenes* unknown.

#### Etymology.

The specific epithet refers to the only known island of occurrence for this species.

#### Distribution.

This new species is known only from a single collection at the type locality in the southern summit region at 870 m on Fatu Hiva, Marquesas Islands.

#### Ecology.

*Coprosma fatuhivaensis* occurs in *Metrosideros collina* windswept wet ridge forest with secondary dominants of *Crossostylis biflora* J. R. Forst. & G. Forst. and *Freycinetia impavida*.

#### Conservation status.

Following the criteria and categories of IUCN (2001) it is assigned a preliminary status of **Critically endangered** (CR): B2a, B2b (i–iii); D: B2: total area of occupancy less than 10 km2 (ca. 5 km2); B2a, a single population known; b (i–iii), habitat continuing decline inferred; D, population estimated to number fewer than 250 individuals (a single individual observed). The suitable habitat for *Coprosma fatuhivaensis* on Fatu Hiva (ca. 85 km2) is indicated as an endangered environment, threatened by feral animals and invasive plants, reducing the extent of the forest. Estimated population size (collector's note) is a single female individual.

**Figure 1.**
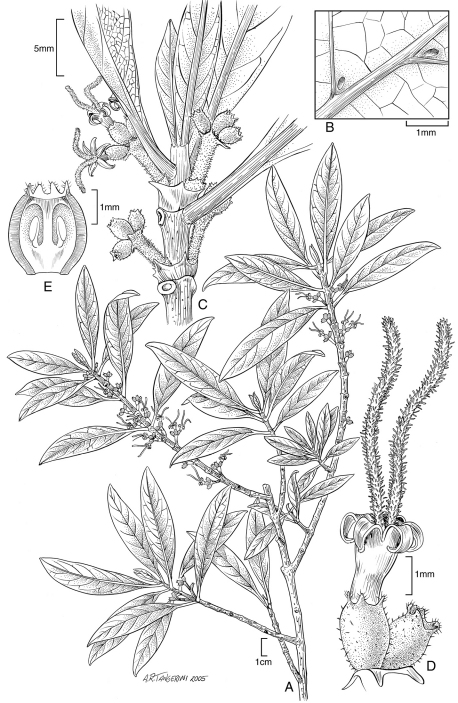
*Coprosma fatuhivaensis* W. L. Wagner & Lorence **A** Flowering branch **B** Lower surface of leaf portion showing domatia **C** Upper stem with female inflorescences **D** Female flowers **E** Longitudinal section of ovary showing basal ovules. Drawn from the type collection (Wood 10137) and field images.

### Coprosma meyeri

W. L. Wagner & Lorence sp. nov.

urn:lsid:ipni.org:names:77112737-1

http://species-id.net/wiki/Coprosma_meyeri

Figs 2 4C, D 

#### Latin.

*Foliis coriaceis 4*–*7.2 longis* × *1.2*–*2 cm latis, oblanceolatis, laminis cum secondaria et altiori obscura venatione, centrali costa solum conspicua, petiolis 0*–*0.1 cm, stipulis ca. 3*–*8 mm longis.*

#### Type.

**Marquesas Islands**: Hiva Oa:Feani area, on Hanamenu trail at summit crest above Vaiumete et Vaiumioi, 1090 m, [9°47.93"S, 139°4.75"W], 30 January 2003, S. Perlman 18337 (Holotype: PTBG!; Isotypes: BISH!, P!, PAP!, US!).

#### Description.

*Shrubs* 2–3 m tall; young stems sparsely villous. *Leaves* opposite, thick-coriaceous, blades 4–7.2 cm × 1.2–2 cm, oblanceolate, only midvein evident, both surfaces glabrous, domatia absent, apex acuminate to acute, base cuneate; petioles ca. 0.1 cm long; stipules 2.5–3 mm long, connate 4/5 of length, both surfaces glabrous, margin dentate with a few conspicuous colleters and ciliate, the colleters usually with a tuft of hairs. *Inflorescences* axillary, apparently trichotomously branched, with very short internodes, with 6(–12) flowers, these 5–6-merous, subsessile. *Flowers*: male flowers with campanulate calyx ca. 2 mm long, the tube 1.5 mm long, the lobes triangular, 0.5 mm long; corolla narrowly funnelform, 5.4–5.7 mm, the tube 3.3 mm long, the lobes 2.1–2.4 mm long, the anthers 2.5–3.1 mm long, filaments to 11 mm long; female flowers unknown. *Fruits and pyrenes* unknown.

#### Distribution.

*Coprosma meyeri* is known only from the type locality at ca. 1090–1113 m elevation in the Feani area along the trail to Hanamenu on the summit crest of Hiva Oa, Marquesas Islands.

#### Ecology.

This new species occurs in cloud-shrouded low wet forest and shrubland dominated by *Metrosideros* and *Weimannia*, with species of *Alsophila*, *Alstonia*, *Ascarina*, *Blechnum*, *Cheirodendron*, *Crossostylis*, *Cyrtandra*, *Dicranopteris*, *Freycinetia*, *Myrsine*, *Oparanthus*, *Psychotria*, *Reynoldsia*, and *Trimenia*.

#### Etymology.

The specific epithet honors Dr. Jean-Yves Meyer, conservation biologist at the Délégation à la Recherche, Polynésie française, in recognition of his untiring efforts to explore and conserve the biodiversity of French Polynesia.

#### Conservation status.

*Coprosma meyeri* is extremely rare, with less than five plants known from a single locality. Following the criteria and categories of IUCN (2001) it is assigned a preliminary status of **Critically Endangered** (CR): B2a, B2b (i-iii); D: B2: total area of occupancy less than 10 km2 (ca. 5 km2); B2a, a single population known; b (i–iii), habitat continuing decline inferred; D, population estimated to number fewer than 250 individuals. The suitable habitat for on Hiva Oa (*c.* 315 km2) is indicated as an endangered environment threatened by human activities (deforestation and fire), feral animals, and invasive plants, reducing the extent of the forest. Its narrow distribution, disturbance from feral pigs and trail clearing, and invasion by alien plant species such as *Syzygium cumini* (L.) Skeels and *Psidium cattleianum* Sabine put it at risk of extinction.

#### Specimen examined.

**Marquesas Islands:** Hiva Oa:Feani area, trail to Hanamenu, along summit crest, 1113 m, [9°48' 3" S, 139°4'682" W], 1 Aug 2005, S. Perlman 19763 (BISH, P, PAP, PTBG, US).

**Figure 2.**
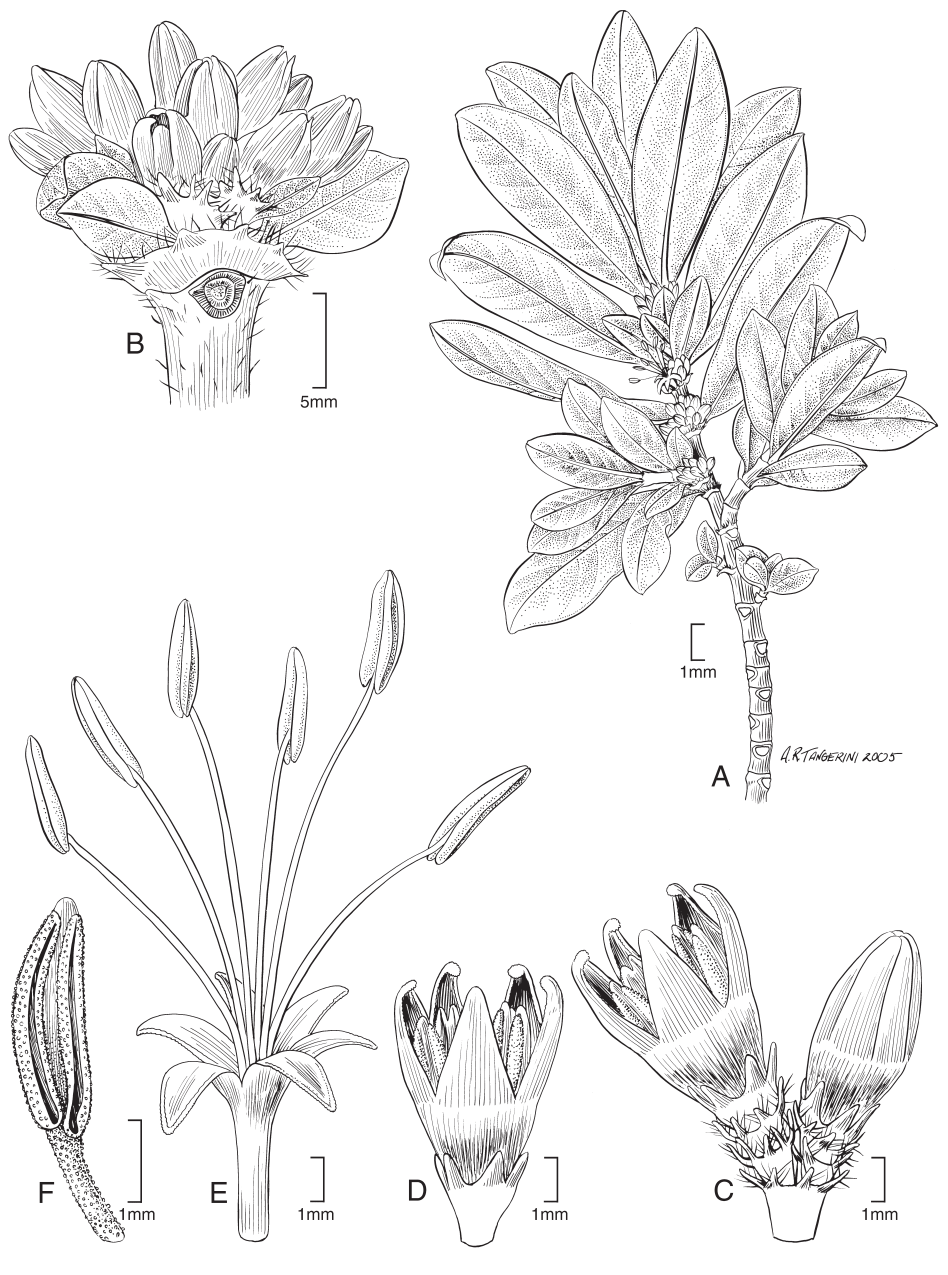
*Coprosma meyeri* W. L. Wagner & Lorence **A** Flowering branch **B** Upper stem with young male inflorescences **C** Male flowers and inflorescence nodes showing the stipules with dentate margin with a few conspicuous colleters and ciliate **D** Young male flower **E** Male flower (corolla and stamens only) **F** Upper part of stamen. Drawn from the type collection (Perlman 18337) and field images.

### Coprosma nephelephila

Florence (Bulletin du Muséum national d'Histoire naturelle, B, Adansonia, Ser. 4, 8: 3, 1986).

http://species-id.net/wiki/Coprosma_nephelephila

#### Type.

**Marquesas Islands**: Nuku Hiva. Route Toovii–Terre Deserte, 6 km apres le col, 5 Juin 1984, 1050 m, J. Florence 6913 (holotype P!; isotypes: BISH!, K!, NY!, PAP!, US!).

#### Description.

*Shrubs or small trees* 2.5–6 m tall; stems glabrous*. Leaves* ternate, chartaceous, blades 5–16.5 × 2–5.5 cm, oblanceolate, oblong or elliptic-oblanceolate, midrib narrow, pinnately veined with 8–13 pairs of secondary veins, both surfaces glabrous, higher level venation conspicuously reticulate, raphides conspicuously visible on lower surface along the major veins, domatia minute, located along secondary veins and sometimes also along midrib near juncture, apex acuminate, base attenuate; petioles 0.3–1.2 cm long, narrowly winged; stipules 4–6 mm long, connate ½–4/5 of length, both surfaces glabrous, margins ciliate and dentate with conspicuous colleters, apex obtuse to a conspicuous appendage. *Inflorescences* axillary, trichotomously branched with 6(–15) flowers, each branch with 1–3 nodes, the uppermost with a 3-flowered cymule, and the others when present with usually only 1–2 flowers developing on each, these 5–7-merous, peduncles 1–2 cm long. *Flowers*: male flowers with calyx campanulate, 2.3–3.7mm long, the tube 0.3–1.2 mm long, the lobes 2–2.5 mm long, corolla 6–7 mm long, the tube and lobes ca. 3–3.5 mm long, stamens 5–8(–12), filaments 7–10 mm long; female flowers with peduncles 0.1–0.7 mm long, calyx tubular, 0.4–0.8 mm long, corolla narrowly funnelform, the tube 0.25–0.4 mm long, the lobes 1.5–2.1 mm long, the styles 7–9 mm long.  *Fruit* 10-11 × 5-6 mm, ellipsoid, apex with persistent calyx teeth. *Pyrenes* narrowly ovoid-ellipsoid, compressed, 10 × 4-4.5 mm, weakly ribbed dorsally.

#### Distribution.

Known only from the Toovii area of Nuku Hiva, Marquesas Islands.

#### Ecology.

*Coprosma nephelephila* is known from 970 to 1100 m elevation, scattered in montane cloud forests dominated by *Metrosideros* and *Weinmannia* associated with with species of *Alsophila*, *Crossostylis*, and *Ilex*.

#### Etymology.

The specific name alludes to its cloud forest habitat preference.

#### Conservation status.

Following the criteria and categories of IUCN (2001) it is assigned a preliminary status of **Endangered** (EN): B1, B2b (i–iii): B1 extent of occurrence <5,000 km²; B2: total area of occupancy less than 500 km² (c. 50 km²); B2b (i–iii), habitat continuing decline inferred in (i) extent of occurrence, (ii) areas of occupancy, and area, (iii) extent and/or quality of habitat. The suitable habitat for *Coprosma nephelephila* on Nuku Hiva (c. 340 km²) is indicated as an endangered environment, threatened by human activity (deforestation and fire), feral animals, and invasive plants, reducing the extent of the forest. The estimated population size for this species is unknown but apparently small.

#### Specimens Examined.

**Marquesas Islands:** Nuku Hiva.Toovii, épaulement S du Mt. Ooumu, 970 m, [08°51'S, 140°08'W], Florence 4342 (BISH, K, NY, P, US); route Toovii-Terre Deserte, km 6.5 après le col, 1010 m, [08°52'S, 140°10'W], Florence 4369 (BISH, P); Haute vallée de Tapueahu, 1070 m, [08°52'S, 140°11'W], Florence 8522 (BISH, CHR, P, PAP, US); summit area of Toovii, near summit of ridge of airport road, S side of new airport road, W side of mountain, 3500 ft [1067 m], Perlman & Wood 15046 (BISH, MO, NY, P, PAP, PTBG, US).

### Coprosma reticulata

Florence (Bulletin du Muséum national d'Histoire naturelle, B, Adansonia, Ser. 4, 8: 6. 1986).

http://species-id.net/wiki/Coprosma_reticulata

#### Type.

**Marquesas Islands**: Nuku Hiva. Toovii, épaulement au-dessus du réservoir [08°52'S, 140°09'W], 970 m, 12 Avril 1982, Florence 4306 (holotype: P!; isotypes: BISH! [2], K!, NY!, PAP!, US!).

#### Description.

*Shrubs or small trees* 3–10 m tall, up to 12 cm diam., stems glabrous. *Leaves* thinly coriaceous, decussate, the blades 8.5–19.7 × 3–13 cm, oblanceolate, oblong or elliptic-oblanceolate, glabrous, pinnately veined with 8–13 pairs of secondary veins, higher level venation conspicuously reticulate, raphides conspicuously visible on lower surface along the major veins, which are dark-colored, domatia small, slightly elongated, located along midrib near juncture with secondary veins, apex acuminate, base attenuate; petioles 0.3–1.3 cm long, narrowly winged; stipules ca. 3–5 mm long, connate ½–4/5 of length, glabrous externally, strigose internally, margins ciliate and dentate with conspicuous colleters, apex obtuse to a conspicuous appendage. *Inflorescences* axillary, simple or occasionally trichotomously branched with 7 flowers, rarely more, each branch with 1–3 nodes the uppermost with a 3-flowered cymule, the others usually with only 1–2 flowers developing on each, these 4–6-merous, peduncles finely, sparsely strigulose. *Flowers:* male flowers with calyx tube 0.5–1.5 mm long, the lobes 0.3–0.9 mm long, the corolla 6.9–8 mm long, the tube 3.5–4.2 mm long, the lobes 3.4–4 mm long, the stamens with filaments 10–13 mm long, the anthers ca. 5 mm long; female flowers with calyx tube 0.4–0.8 mm long, the lobes 0.3–0.6 mm long, corolla narrowly funnelform, the tube 1.5–2 mm long, the lobes 2.2–2.5 mm long, the styles 8–10 mm long. *Fruit* 7–8 × 3.5–4 mm, obovoid to ellipsoid, ripening orange, apex with persistent calyx teeth. *Pyrenes* ovoid-ellipsoid, compressed, 6 × 3 mm, rugulose, dorsally weakly 1-ribbed.

#### Distribution.

Known only from the Toovii area of Nuku Hiva, Marquesas Islands, at elevations from 800 to 1100 m.

#### Ecology.

*Coprosma reticulata* occurs in wet forest and cloud forest habitat with species of *Alsophila*, *Crossostylis*, *Fagraea*, *Freycinetia*, *Metrosideros*, *Weinmannia*, and an understory of ferns including the genera *Dicranopteris*, *Histiopteris*, and *Nephrolepis*.

#### Etymology.

The specific epithet refers to the distinctive reticulate network of tertiary veins.

#### Conservation status.

Based on the IUCN criteria and categories this species is assigned a preliminary Red List status of **Endangered** (EN): B1, B2b (i–iii): B1 extent of occurrence <5,000 km²; B2: total area of occupancy less than 500 km² (c. 50 km²); B2b (i–iii), habitat continuing decline inferred in (i) extent of occurrence, (ii) areas of occupancy, and area, (iii) extent and/or quality of habitat. The suitable habitat for *Coprosma reticulata* on Nuku Hiva (c. 340 km²) is indicated as an endangered environment, threatened by human activity (deforestation and fire), feral animals, and invasive plants, reducing the extent of the forest. The population size for this species is unknown, but based on available herbarium specimens (14) it is apparently not as rare as its Marquesan congeners.

#### Specimens Examined.

**Marquesas Islands**: Nuku Hiva.Toovii, vallon au-dessus du réservoir, 805 m, [08°52'S, 140°09'W], Florence 4316 (BISH, K, NY, P, PAP, US); Toovii, épaulement au-dessus du réservoir, 910 m, [08°52'S, 140°09'W], Florence 4325 (BISH, P); Toovii, épaulement S du Mt. Ooumu, 980 m, [08°51'S, 140°08'W], Florence 4346 (BISH, K, NY, P, US); Toovii, flanc W de la vallée de la Tapuaooa, 925 m, [08°51'S, 140°09'W], Florence 7452 (BISH, P, PAP); Toovii region, trail along ridge from near l'Economie Rurale complex to Ooumu peak, 860–1080 m, Lorence et. al. 6116 (BISH, PAP, PTBG, US); Mt. Ooumu, 1066 m, Mumford & Adamson 582(BISH); Toovii Plateau, trail behind l'Economie Rurale, toward Ooumu peak, 3100 ft, Perlman 10124 (BISH, MO, PAP, PTBG, US); off new airport road, W of summit crest, W of Peak #1227 M., drainages of Matatekouaehi, 3580 ft, Perlman & Wood 15041 (BISH, PAP, PTBG, US); along old airport road on W side of summit ridge, W of Toovii, 1.5 miles S of new airport road, 3360 ft, Perlman & Wood 15067 (AD, BISH, MO, NY, P, PAP, PTBG, US); Toovii, 850 m, Thibault 127 (BISH, P, US); Ooumu area, top of Tapueahu Valley off new hwy, 3500–3700 ft [1067–1178 m], [08°51'53"S, 140°10'63"W], Wood et. al. 6344 (PTBG), [08°51'S, 140°19'W], Wood & Perlman 4587 (PTBG, US), Wood & Perlman 4627 (BISH, MO, PAP, PTBG, US).

### Coprosma temetiuensis

W. L. Wagner & Lorence sp. nov.

urn:lsid:ipni.org:names:77112738-1

http://species-id.net/wiki/Coprosma_temetiuensis

Figs 3 4E, F 

#### Type.

**Marquesas Islands**: Hiva Oa.Along trail from Atuona to Mt. Temetiu, moist forest, 700 m, 29 January 2003, D. H. Lorence, L. Dunn, & J. Price 8931(Holotype: PTBG!; Isotypes: BISH!, P!, PAP!, US!).

*Foliis tenuiter coriaceis, 5.1*–*9.1* × *2.1*–*3.6 cm, petiolis 0.3*–*0.8 cm longis, stipulis 1.5*–*3 mm longis.*

#### Description.

*Shrubs* 4–6 m tall; young stems short-pilose. *Leaves* opposite, thinly coriaceous, blades 5.1–9.1 × 2.1–3.6 cm, elliptic, pinnately veined with 8–9 pairs of veins, higher level venation conspicuously reticulate, both surfaces glabrous, domatia small, usually somewhat elongate, located along midrib near juncture of secondary veins, apex acuminate, base cuneate; petioles 0.3–0.8 cm long; stipules ca. 1.5–3 mm long, connate 4/ 5 of length, both surfaces glabrous, margins glabrous and dentate with a few small colleters, apex obtuse to a conspicuous appendage. *Inflorescences* axillary with 3(–7) flowers, with 1–3 nodes, the uppermost with a 3-flowered cymule, the others with usually only 1–2 flowers developing on each, these 5-merous, the peduncles 0–4 mm long, finely short-pilose. *Flowers: male flowers* unknown; *female flowers* with calyx short-tubular, 0.25–0.3 mm long, the teeth ca. 0.2 mm long, glabrous, corolla narrowly funnelform, the tube 0.3 mm long, the lobes 2.2–2.5 mm long, glabrous, the styles 5.5–7 mm long. *Young fruits* sparsely pilose, fruits and pyrenes otherwise unknown.

#### Distribution.

Known only from areas the vicinity of Mt. Temetiu, the highest peak on Hiva Oa, Marquesas Islands.

#### Ecology.

*Coprosma temetiuensis* occurs at 700–880 m elevation in mesic forest and cloud forest with *Metrosideros* and *Weimannia*, associated with species of *Alsophila*, *Crossostylis*, *Freycinetia*, *Hibiscus*, *Pandanus*, and *Phyllanthus*.

#### Etymology.

The specific epithet refers to the only known locality for this species.

#### Specimen examined.

**Marquesas Islands**: Hiva Oa.Hanamenu region, up Hanamenu valley to the drainages below and west of Temetiu, 884 m, [9°76'S, 139°0'W], Wood 10239 (PTBG).

#### Conservation status.

This species is extremely rare, with only two plants known from this locality. Following the criteria and categories of IUCN (2001) it is assigned a preliminary status of **Critically Endangered** (CR): B2a, B2b (i–iii); D: B2: total area of occupancy less than 10 km2 (ca. 5 km2). B2a, a single population known; b (i–iii), habitat continuing decline inferred; D, population estimated to number fewer than 250 individuals. The suitable habitat for *Coprosma temetiuensis* on Hiva Oa (*c.* 315 km2) is indicated as an endangered environment, threatened by human activities (deforestation and fire), feral animals, and invasive plants, reducing the extent of the forest.

**Figure 3.**
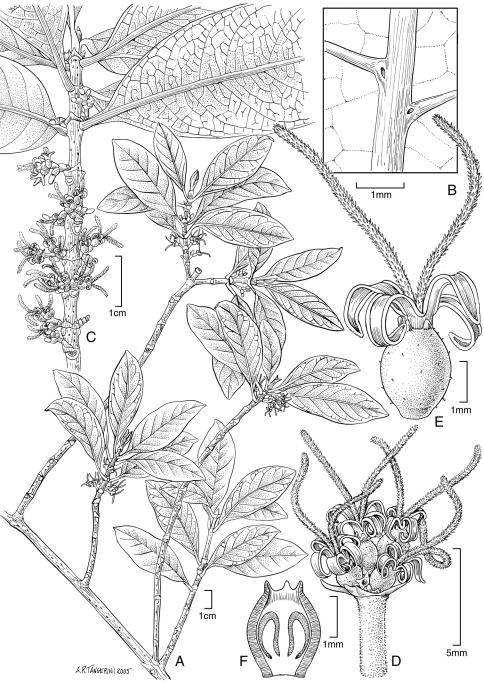
*Coprosma temetiuensis* W. L. Wagner & Lorence **A** Flowering branch **B** Lower surface of leaf portion showing domatia **C** Upper stem with female inflorescences **D** Female inflorence **E** Female flower **F** Longitudinal section of developing fruit showing young basal seeds. Drawn from the type collection (Lorence et al. 8931) and field images.

**Figure 4.**
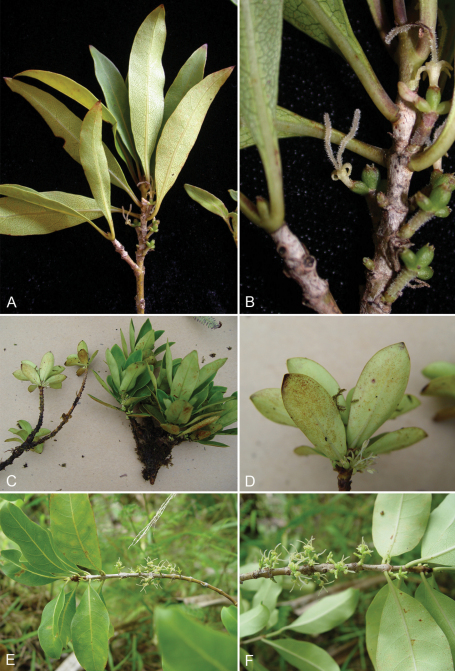
Three new Marquesan *Coprosma* species. *Coprosma fatuhivaensis* W. L. Wagner & Lorence **A, B** branchlets with female flowers (Fatu Hiva, Wood 10137, photos K.R. Wood); *Coprosma meyeri* W. L. Wagner & Lorence **C, D** branchlets with male flowers (Hiva Oa, Perlman 18337, photos D. Lorence); *Coprosma temetiuensis* W. L. Wagner & Lorence **E, F** branchlets with female flowers(Hiva Oa, Lorence & Dunn 8931, photos D. Lorence).

## Supplementary Material

XML Treatment for Coprosma

XML Treatment for Coprosma esulcata

XML Treatment for Coprosma fatuhivaensis

XML Treatment for Coprosma meyeri

XML Treatment for Coprosma nephelephila

XML Treatment for Coprosma reticulata

XML Treatment for Coprosma temetiuensis

## References

[B1] Anderson CL Rova JHE Andersson L (2001) Molecular Phylogeny of the Tribe Anthospermeae (Rubiaceae): Systematic and Biogeographic Implications. Australian Systematic Botany 14: 231-244 doi:10.1071/SB00021

[B2] Brown FBH (1935) Flora of southeastern Polynesia. III. Dicotyledons. Bernice P. Bishop Museum Bulletin 130: 1-386

[B3] Florence J (1986) Sertum polynesicum II. Rubiaceae nouvelles des Îles Marquises (Polynésie Française). Bulletin du Muséum national d'Histoire naturelle, Paris, sér. 4, 8, section B, Adansonia 1: 3–11

[B4] Fosberg FR (1939) *Psychotria* (Rubiaceae) in the Marquesas Islands. Notulae Systematicae (Paris) 8 (3): 161-173

[B5] Fosberg FR (1956) Studies of Pacific Rubiaceae I–IV. Brittonia 8: 165-178 doi:10.2307/2804734

[B6] Heads MJ (1996) Biogeography, taxonomy and evolution in the Pacific genus *Coprosma* (Rubiaceae). Candollea 51: 381-405

[B7] Oliver WRB (1935) The genus *Coprosma*. Bernice P. Bishop Museum Bulletin 132: 1–207, plates 1–59.

[B8] Wagner WL Lorence DH (1997) Studies of Marquesan vascular plants: Introduction. Allertonia 7: 221-225

